# CDC's Male Circumcision Recommendations Represent a Key Public Health Measure

**DOI:** 10.9745/GHSP-D-16-00390

**Published:** 2017-03-24

**Authors:** Brian J Morris, John N Krieger, Jeffrey D Klausner

**Affiliations:** a School of Medical Sciences and Bosch Institute, University of Sydney, Sydney, New South Wales, Australia.; b University of Washington School of Medicine and VA Puget Sound Health Care System, Section of Urology, Seattle, WA, USA.; c Department of Epidemiology, Jonathan and Karin Fielding School of Public Health, University of California, Los Angeles, CA, USA.

## Abstract

Frisch and Earp, opponents of male circumcision, have criticized draft recommendations from the CDC that advocate counseling men and parents of newborn boys in the United States about the benefits and risks of male circumcision. We provide a rebuttal to Frisch and Earp's criticisms and contend that the recommendations are entirely appropriate and merit consideration for policy development.

## INTRODUCTION

After an extensive evaluation of the scientific evidence, the United States Centers for Disease Control and Prevention (CDC) released draft policy recommendations in December 2014 affirming male circumcision (MC) as an important public health measure.^1^^–^^3^ The CDC's summary^1^ (Box 1) was accompanied by a 61-page literature review.^2^ The CDC supported the 2012 American Academy of Pediatrics (AAP) infant MC policy^4^^,^^5^ (Box 2) and recommended that providers: (1) give parents of newborn boys comprehensive counseling about the benefits and risks of MC; (2) inform all uncircumcised adolescent and adult males who engage in heterosexual sex about the significant, but partial, efficacy of MC in reducing the risk of acquiring HIV and some sexually transmitted infections (STIs) through heterosexual sex, as well as about the potential harms of MC; and (3) inform men who have sex with men (MSM) that while it is biologically plausible that MC could benefit MSM during insertive sex, MC has not been proven to reduce the risk of acquiring HIV or other STIs during anal sex.^3^

BOX 1 **U.S. Centers for Disease Control and Prevention's Summary of Its Draft Male Circumcision Recommendations^1^**

These recommendations are intended to assist health care providers in the United States who are counseling men and parents of male infants, children and adolescents in decision-making about male circumcision. Such decision-making is made in the context of not only health considerations, but also other social, cultural, ethical, and religious factors. Although data have been accumulating about infant male circumcision for many years, clinical trials conducted between 2005–2010 have demonstrated safety and significant efficacy of voluntary adult male circumcision performed by clinicians for reducing the risk of acquisition of human immunodeficiency virus (HIV) by a male during penile-vaginal sex (“heterosexual sex”). Three randomized clinical trials showed that adult male circumcision reduced HIV infection risk by 50–60% over time. These trials also found that adult circumcision reduced the risk of men acquiring two common sexually transmitted infections (STIs), herpes simplex virus type-2 (HSV–2) and types of human papilloma virus (HPV) that can cause penile and other anogenital cancers, by 30%. Since the release of these trial data, various organizations have updated their recommendations about adult male and infant male circumcision.

BOX 2 **Conclusions of the 2012 Circumcision Policy Statement by the American Academy of Pediatrics Task Force on Circumcision^3^**

Systematic evaluation of English-language peer-reviewed literature from 1995 through 2010 indicates that preventive health benefits of elective circumcision of male newborns outweigh the risks of the procedure. Benefits include significant reductions in the risk of urinary tract infection in the first year of life and, subsequently, in the risk of heterosexual acquisition of HIV and the transmission of other sexually transmitted infections.

The procedure is well tolerated when performed by trained professionals under sterile conditions with appropriate pain management. Complications are infrequent; most are minor, and severe complications are rare. Male circumcision performed during the newborn period has considerably lower complication rates than when performed later in life.

Although health benefits are not great enough to recommend routine circumcision for all male newborns, the benefits of circumcision are sufficient to justify access to this procedure for families choosing it and to warrant third-party payment for circumcision of male newborns. It is important that clinicians routinely inform parents of the health benefits and risks of male newborn circumcision in an unbiased and accurate manner.

Parents ultimately should decide whether circumcision is in in the best interests of their male child. They will need to weigh medical information in the context of their own religious, ethical, and cultural beliefs and practices. The medical benefits alone may not outweigh these other considerations for individual families.

Findings from the systematic evaluation are available in the accompanying technical report. The American College of Obstetricians and Gynecologists has endorsed this statement.

The CDC has a mandate to use the best available evidence to inform the public on interventions for disease prevention. In the case of early infant MC, there are few public health interventions in which the scientific evidence in favor is now so compelling. Despite this, opponents of MC do not accept the CDC's position. Two prominent opponents, Frisch and Earp, published arguments that led them to conclude that “from a scientific and medical perspective, current evidence suggests that circumcision is not an appropriate public health measure for developed countries such as the United States.”^6^

Here, we critically assess the evidence used by Frisch and Earp to support their thesis and respond to their main criticisms (summarized in Box 3).

BOX 3 **Criticisms of the U.S. Centers for Disease Control and Prevention's Draft Male Circumcision Recommendations and Responses**

In a recently published article, Frisch and Earp^6^ oppose the 2014 draft MC recommendations from the U.S. Centers for Disease Control and Prevention (CDC),^3^ referring to what they believe are “numerous scientific and conceptual shortcomings.” Here, we quote these 7 criticisms by Frisch and Earp and provide our response to each criticism.

1. *Failure to provide a thorough description of the normal anatomy and functions of the penile structure being removed at circumcision (i.e., the foreskin)*Response: There seems to be no need for the CDC to provide a thorough description of the anatomy and functions of the foreskin.
2. *Failure to consider the intrinsic value to some men of having an unmodified genital organ*Response: While some men may believe there is “an intrinsic value to having an unmodified genital organ,” those men should be made aware of the risks posed by their foreskin.
3. *Undue reliance on findings from sub-Saharan Africa concerning circumcision of adult males (as opposed to infants or children)*Response: The evidence shows the CDC is correct in concluding that findings from sub-Saharan Africa concerning circumcision of adult males for protection against heterosexually-acquired HIV and certain other STIs also apply to men in the United States. The findings also apply to boys when they grow up. Moreover, the cumulative lifetime benefit is greatest if circumcision is performed early in infancy since early infant circumcision is simpler, more convenient, and carries lower risk than when performed later, and circumcision confers immediate protection against urinary tract infections, phimosis, balanitis, and, when older, specific STIs and genital cancers. MC also protects the female partners, as confirmed in randomized controlled trials.
4. *Uncritical reliance on a prima facie implausible benefit-risk analysis performed by a self-described circumcision advocate*Response: The benefit-risk analysis used by the CDC is based on the best current evidence relevant to the United States, and the results are plausible.
5. *Reliance on misreported statistics to downplay the problem of pain in the youngest of boys*Response: While procedural pain can occur during circumcision, the evidence cited by the CDC indicates that, with use of local anesthetic, pain is negligible in the first week of a boy's life. Frisch and Earp misconstrue pain statistics to overplay the issue of pain.
6. *Reliance on incomplete register data to assess the frequency of short-term post-operative complications associated with circumcision, leading to a likely underestimation of their true frequency*Response: By selective citation and misrepresentation of findings, Frisch and Earp overstate the frequency of short-term postoperative complications associated with MC while ignoring data from large high-quality studies such as those published recently by CDC researchers.
7. *Serious underestimation of the late-occurring harms of circumcision presenting months to years after the operation (most notably meatal stenosis).*Response: Frisch and Earp selectively cite small, outdated, weak studies, often involving traditional circumcisers, and misrepresent data while ignoring large, high-quality studies. As a result, they overestimate the frequency of meatal stenosis occurring years after the MC procedure.

## BENEFITS VERSUS RISKS

MC confers immediate and lifelong protection against numerous medical conditions (Box 4).^1^^,^^2^^,^^4^^,^^5^^,^^7^^–^^9^ For example, MC protects against a number of STIs including HIV, and it partially protects against oncogenic types of human papillomavirus (HPV)^10^^–^^15^ that together with phimosis, balanitis, and smegma are major risk factors for penile cancer,^10^^,^^16^^–^^18^ as shown in meta-analyses that found 12-, 4-, and 3-fold statistically significant higher risks of penile cancer for phimosis, balanitis, and smegma, respectively.^16^ Infancy is the ideal time for MC and there are cogent reasons why it should not be delayed until the boy or man can make up his own mind^19^ (Table).

**TABLE.** Why Infant Male Circumcision Is Preferable to Male Circumcision at a Later Age

Infant Male Circumcision	Male Circumcision of Older Boys and Men
Simple	More complex
Quick (a few minutes)	Takes half an hour or more
Low cost	Expensive (often unaffordable)
Low risk (adverse events 0.4%)^20^	Moderate risk (adverse events 4%–8%)^20^
Bleeding is minimal	Bleeding more common, requiring cautery or other interventions
No need for sutures	Sutures or tissue glue needed
Convenient (baby mostly sleeps)	Inconvenient (time off school or work required)
Local anesthesia for those <2 months of age^21^	General anesthesia for those >2 months to 9 years of age; local anesthesia for men, although general anesthesia sometimes preferred by surgeon
Healing is fast (2 weeks)^21^	Healing takes 6 weeks or more
Cosmetic outcome usually good	Stitch marks may be seen
No long-term memory of procedure	Fear of undergoing an operation
	Abstinence from sexual intercourse for the 6-week healing period

Male circumcision confers immediate and lifelong protection against numerous medical conditions.

BOX 4 **Medical Conditions That Male Circumcision Protects Against Over the Lifetime**

- Urinary tract infection
- Penile inflammation, for example, balanitis, balanoposthitis, lichen sclerosus
- Candidiasis
- Phimosis and paraphimosis
- Inferior hygiene
- Sexually transmitted infections including high-risk human papillomavirus (HPV), genital herpes simplex virus (HSV), trichomoniasis, mycoplasma, syphilis, chancroid, and HIV
- Physical injuries to the foreskin, including coital injuries
- Cancers of the penis, prostate, and cervix

Sources: CDC technical review^2^ and draft policy recommendations,^3^ AAP review^5^ and infant MC policy statement,^4^ risk-benefit analyses by Morris et al.^7^^–^^9^

Disputing the value of MC's protection against STIs, Frisch and Earp argue that less invasive STI prevention strategies should instead be promoted, such as encouraging safe sex practices. But we argue that public health messages normally include *all* effective measures for protection against disease, and in the case of STIs, MC complements current safe sex messages. The effectiveness of each approach should, moreover, be considered in real-world settings.

Frisch and Earp also contend that many STIs can be treated effectively if they do occur. We dispute that logic and instead argue that prevention is preferable to treatment, especially for viruses for which there is no cure (e.g., HIV, herpes simplex virus [HSV], and HPV). And for bacterial STIs and urinary tract infection (UTI), antibiotic-resistant strains mean that infections that were once easily treatable can now be life threatening.^22^^–^^25^

The benefits of medical procedures should always, of course, be weighed with the potential risks. Frisch and Earp question whether the potential benefits of MC are “worth” the risk, pointing to potential risks of surgical accidents and supposed adverse psychological or sexual effects. The risk of major surgical mishaps with MC, however, is extremely low and the benefits gained from MC far exceed risks.^2^^,^^5^^,^^8^^,^^9^ Furthermore, there is no long-term adverse effect of infant MC on psychological^26^^,^^27^ or sexual^28^^–^^33^ outcomes. Systematic reviews have found no adverse effect of MC on sexual function,^28^^,^^29^^,^^33^ sensitivity, or satisfaction.^29^ A meta-analysis of all common male sexual dysfunctions found none were related to MC status.^28^ Furthermore, the third British National Survey of Sexual Attitudes and Lifestyles (Natsal-3), a large national probability survey, which used a new, comprehensive, validated measure of sexual function, the Natsal-SF, presented findings for 6,293 men and 8,869 women aged 16–74 years, broadly representative of the British population. The survey concluded that MC is not associated with men's overall sexual function.^31^ In addition, a recent survey of 1,000 adults by an Internet-based market research firm that is a member of the British Polling Council found 29% of *un*circumcised men wished they had been circumcised, compared with only 10% of circumcised men who wished they had not been circumcised (margin of error ±4%).^34^ A randomized controlled trial (RCT) of uncircumcised men in Kenya found sexual pleasure increased in most men after MC.^35^ It is possible some circumcised men may be unhappy due to exposure to misleading propaganda that dominates the Internet.

The benefits gained from male circumcision far exceed risks.

A risk-benefit analysis^8^ cited by the CDC^2^ found that benefits of infant MC exceed risks by over 100:1. A letter^36^ questioning this risk-benefit analysis that Frisch and Earp cite contained misunderstandings, as pointed out in the response to the letter.^37^ A large study by CDC researchers found frequency of adverse events for newborn MC was 0.4%.^20^ These data are robust and withstand Frisch and Earp's non-evidence-based speculation to the contrary. Frisch and Earp refer to analysis conducted by the Canadian Paediatric Society (CPS) that tabulated risks and benefits of newborn MC, concluding the risk-benefit ratio was “closely balanced.”^38^ But risk figures in the CPS analysis were exaggerated because they were drawn from a global study that included data from traditional (non-medical) MCs^39^ and outlier studies while ignoring a more recent study by CDC researchers of 1.4 million (mostly newborn) MCs.^20^ In addition, multiple benefits (phimosis, balanitis, balanoposthitis, prostate cancer, some STIs, candidiasis, and lifetime prevalence of urinary tract infections) were omitted, and the actual risk-benefit ratio was not determined. (See critique^40^ for further details.)

In addition to potential risks from the surgical procedure, Frisch and Earp point to other potential negative consequences of MC, namely, “the loss of healthy, functional tissue” (i.e., the foreskin). But they fail to acknowledge that the healthy foreskin of an uncircumcised male remains vulnerable to adverse medical conditions, infections, and genital cancers. We draw attention to a Danish study by Sneppen and Thorup that found “significant morbidity related to foreskin problems in a predominantly uncircumcised population.”^41^ It pointed out that the reason most Danish boys might go through infancy, childhood, and adolescence without being circumcised reflects “the strict foreskin-preserving culture of Denmark.”^41^ “More than 5% … were admitted to the pediatric surgical department with foreskin-related problems [mainly phimosis] and at least 1.66% of the boys needed surgical procedures in [*sic*] general anesthesia.”^41^ Of these, 24% initially received a circumcision and another 5% received circumcision after alternative treatment failed. Moreover, foreskin-preserving preputioplasty had to be repeated in 5.5% of cases (repeat surgery for MC was lower, at 2%), further exposing the boy to surgical risks.

The uncircumcised foreskin remains vulnerable to adverse medical conditions, infections, and genital cancers.

Foreskin problems continue into adulthood, as does MC for medical and cosmetic reasons. Since some men might not seek medical attention, especially for sexual or genital conditions, foreskin problems will always be more common than evident in case studies such as the one by Sneppen and Thorup. Infant MC would prevent later foreskin problems and obviate the need for later MC which is more costly and risky.^19^ Risk-benefit analyses calculated that half of uncircumcised males will, over their lifetimes, suffer from an adverse medical condition attributable to their foreskin.^8^^,^^42^ One such condition among uncircumcised men is lichen sclerosus (a condition that creates patchy, white skin that is thinner than normal, most often affecting the genital area).^43^ This had a prevalence of 0.37% in the Danish study.^41^ Lichen sclerosus is difficult to treat, and treatment has a low success rate.^43^ Frisch and Earp go into a lengthy argument that another condition, meatal stenosis (a subcategory of urethral stricture disease), is one of the most common complications after MC, citing numerous studies. But most of the studies they cite to support their claim are small, quite old, comprised of MC performed by non-medical personnel, lack a control group of uncircumcised males, and either include no statistical analyses or include *P* values that were not statistically significant. Furthermore, meatal stenosis is seen in *uncircumcised* males as well. In the Danish study, risk of developing meatal stenosis in uncircumcised boys before 18 years of age was 0.17%.^41^ Prevalence was 0.01% in a large U.S.^20^ study of infants and a U.K.^44^ study of boys aged 0–15 years, although follow-up in each study was only 6 months. Among the lichen sclerosus patients in the Danish study, 37.5% developed meatal stenosis.^40^

Finally, Frisch and Simonsen reported that circumcised boys may be at increased risk for autism spectrum disorder (ASD) due to MC-related pain.^45^ Their conclusion was based on their finding of ASD prevalence of 6.3% in circumcised and 1.5% in uncircumcised Danish boys. That report has been criticized.^41^^,^^46^^,^^47^ Sneppen and Thorup, in particular, found ASD prevalence was 7.2% in *uncircumcised* Danish boys and suggested Frisch's study suffered from confounding.^41^

## DOES AN “INTACT SEXUAL ORGAN” HAVE ANY VALUE?

Frisch and Earp criticize (without *scientific* evidence) the CDC's draft recommendations for not discussing the “protective and sexual functions” of the foreskin. A study by Frisch claiming sexual dysfunctions in circumcised men^48^ was one-sided and suffered from confounding and statistical flaws.^29^^,^^49^ In this Danish study, MC of the mostly (89%) Lutheran or non-religious Danish men surveyed was likely for medical conditions that often affect sexual function, either directly or from a preexisting psychological aversion that develops because the condition causes difficulties with intercourse.^29^^,^^49^^,^^50^ Participation bias, small sample sizes for cases among the 5% who were circumcised, and failure to correct for multiple testing were also noted.^29^^,^^49^ Confounding and statistical flaws were also noted for a study of penile sensitivity by Sorrells et al.^51^ That study was severely criticized for a multitude of reasons, including failure to correct for multiple testing that, if performed, would have rendered the age-adjusted *P* value of .014 non-significant; mode of recruitment; large discrepancies in subject numbers between the methods and results sections; and failure to compare comparable sites on the penis of circumcised and uncircumcised men (which when performed by the critics were shown to be not statistically significant).^29^^,^^52^ A recent Canadian study concluded that “if sexual function is related to circumcision status, this relationship is not likely the result of decreased penile sensitivity stemming from neonatal circumcision.”^32^ It has also been found that sensory nerve endings (Meissner's corpuscles) in the foreskin are lower in density and smaller in size than those in other glabrous (hairless) epithelia of the body.^53^

Sensitivity to vibration (not tested by either Bossio et al.^32^ or Sorrells et al.^51^) correlates with sexual response and is similar in uncircumcised and circumcised men.^30^ Studies of histological correlates of sexual sensation concluded that the glans, not the foreskin, is involved in sexual sensation.^30^^,^^54^ C-fibers (activated by thermal stimuli and punctate pain) may be involved in erotic sensation and sexual arousal.^55^ Similar unmyelinated free nerve endings predominate in the glans, not the foreskin.^30^

Thus, speculation and outdated opinion pieces claiming special properties of the foreskin, such as in penile function and masturbation, should be viewed with skepticism. Perhaps sensitivity of the foreskin to fine touch (which activates Aβ, large diameter, myelinated nerve fibers) might have served as an “early warning system” in our naked upright forebears from the intrusion of biting insects and parasites while protecting the glans.^56^

The area of the outer and inner foreskin combined spans a wide range: 7–100 cm^2^ (n=965)^57^ and 18–68 cm^2^ (n=8),^58^ respectively. In discussing vestigial structures, Charles Darwin stated, “An organ, when rendered useless, may well be variable, for its variations cannot be checked by natural selection.”^59^ The variability in foreskin size is consistent with the foreskin being a vestigial structure. Larger foreskins place uncircumcised men at increased risk for HIV infection.^56^

## SCIENTIFIC INFERENCE FROM AFRICAN TRIALS

Arguments by MC opponents disputing the validity of the large African RCTs showing that MC provides substantial protection against heterosexually-acquired HIV infection have been exposed as fallacious.^60^^–^^72^ Frisch and Earp instead question the CDC for applying the African trial findings to the United States. Although the proportion of HIV infections acquired heterosexually in the United States is far less than in sub-Saharan Africa, in some U.S. localities heterosexually-acquired HIV incidence is high. Furthermore, 2014 CDC figures show 24% of new HIV infections in the United States involved heterosexual contact.^73^ It was estimated that if all boys in the 2011 annual U.S. male birth cohort were circumcised, 5,530 HIV infections would be prevented over their lifetime.^74^ Lifetime risk of HIV diagnosis in heterosexual males in the United States is currently 1 in 524.^75^ The increase in HIV infections in African-Americans, in particular, has been faster than in all other groups.^76^ Modeling by the CDC found MC could reduce heterosexual HIV risk by approximately 21% in African-Americans and by approximately 12% in Hispanics, and costs would be saved in each group.^77^ Actual MC-related risk reduction in heterosexual African-American men with known HIV exposure was 51%.^78^

Male circumcision could reduce heterosexual HIV risk by about 21% in African-Americans and 12% in Hispanics.

Comparison of HIV and MC prevalence in high-income countries also suggest MC has a protective effect, providing further support to the applicability of the African MC trials to the United States and other high-income countries. For example, HIV prevalence in the mostly uncircumcised populations of France and the Netherlands was much higher than in Israel where almost all men are circumcised, despite all other risk factors being comparable.^79^ In Australia, where MC is less common than in the United States and Israel, the number of HIV infections related to heterosexual contact has increased by 28% over the past decade, representing 25% of new diagnoses in 2013, 29% being in Australian-born patients.^80^ In Canada, where infant MC prevalence has, like in Australia, declined in recent decades, 9.5% of new HIV infections involve men infected heterosexually.^81^

As well as substantial protection against HIV, data from the African RCTs reinforced the ability of MC to protect against several other STIs in heterosexual males,^10^^,^^11^^,^^13^^,^^16^^,^^71^^,^^82^^–^^90^ as well as their female sex partners^10^^,^^91^^–^^95^ and among MSM who are insertive-only.^96^^–^^100^ With regard to MSM in particular, a Cochrane analysis of MC and HIV prevalence among MSM found results were statistically significant among 3,465 men in 7 studies reporting an insertive role (odds ratio, 0.27; 95% confidence interval, 0.17 to 0.44; I^2^=0%), but were not significant among 1,792 men in 3 studies reporting a receptive role (odds ratio, 1.20; 95% confidence interval, 0.63 to 2.29; I^2^ = 0%).”^1^^,^^80^ MC also reduces the risk of potentially fatal penile, prostate, and cervical cancer.^10^^,^^16^^–^^18^^,^^101^^–^^104^ Partial protection against prostate cancer incidence was seen in U.S.^101^ and Canadian^103^ studies and in a meta-analysis of all studies,^104^ the protective effect being strongest (36%^101^ and 60%^103^) in North American men of African heritage.

## EUROPEAN EXPERIENCE

It is misleading to compare HIV prevalence in the United States, where MC is common, with a similar or slightly lower prevalence in Europe, where MC is uncommon, and conclude that MC does not make a difference, as Frish and Earp do. Unlike Africa, most HIV infections in the United States and Europe occur in MSM. HIV subtype B arrived in Haiti from Africa between 1961 and 1970, reaching the United States in the mid-1970s after Haiti became a popular destination for sex tourism.^105^ The United States thus had a “head-start” on Europe and the rest of the developed world.

## COSTS

Because Frisch and Earp dispute the low prevalence of adverse events with MC, they disagree with the conclusions from a cost-benefit study by authors from the Johns Hopkins University.^74^ This study found that if infant MC prevalence in the United States decreased from the current 80% prevalence^106^ to the levels of 10% typical in Europe, the additional direct medical costs in infancy and later for treatment of UTIs and STIs would exceed US$4.4 billion over 10 annual birth cohorts, after accounting for the cost of the MC procedure and treatment of MC complications.^74^ If early infant MC rates decreased to 10%, lifetime prevalence of infant UTIs would increase by 211.8%, high- and low-risk human HPV by 29.1%, HSV-2 by 19.8%, and HIV by 12.2%.^74^ Among females, lifetime prevalence of bacterial vaginosis would increase by 51.2%, trichomoniasis by 51.2%, high-risk HPV by 18.3%, and low-risk HPV by 12.9%.^74^

Frisch and Earp also take issue with the CDC's modeling findings^77^ that MC in the United States was cost-saving for HIV prevention among black and Hispanic males but not necessarily among, what Frisch and Earp refer to as, “the majority population of white males.” The CDC found that “for all males, circumcision resulted in undiscounted lifetime HIV-related health care savings of $2,070 per male and discounted lifetime HIV-related health care savings of $427.”^77^ As pointed out in the CDC study, the lack of cost-effectiveness for white males may be because white males in the United States already have a high prevalence of MC, a low lifetime risk of HIV, and a low risk of acquiring HIV through heterosexual sex compared with black and Hispanic males. We also contend that if other factors were considered in the model, including medical conditions associated with lack of MC, infections and genital cancers in both sexes, and indirect costs, MC would likely be cost-saving among U.S. whites as well. For example, in the absence of MC in the United States, there would be 24%–40% more prostate cancer cases and US$0.8–1.1 billion extra in costs for treatment and terminal care per year.^107^ Annual cost-savings for genital cancer prevention by a shift from the current rate of 10%–20% for infant MC in Australia to 80% was calculated as $1–2 million for direct medical costs, unadjusted for inflation.^108^

The U.S. state of Florida provides an illustrative case study of the cost-savings benefits of MC. In 2003, the state withdrew Medicaid health insurance coverage for infant MC. That resulted in a 6-fold increase in medical costs for publicly funded MCs for medical indications, because later MCs are substantially more expensive than early infant MCs.^109^ In response, Florida restored Medicaid coverage in 2014.

Thus, in contrast to the assertions by Frisch and Earp, the cost-savings estimated by the CDC^77^ and Johns Hopkins researchers^74^ appear conservative. Moreover, cost-savings from infant MC apply to whites, blacks, and Hispanics.

## PROCEDURAL AND POST-OPERATIVE PAIN IN INFANTS

Claims of long-term psychological, emotional, and sexual impediments from infant MC “pain” are anecdotal.^110^^,^^111^ In contrast, in a longitudinal study of New Zealand boys circumcised in 1977, MC had no adverse effect on breastfeeding outcomes or cognitive ability later in childhood.^26^ In another follow-up study, of Swedish boys after MC, the boys showed no adverse psychological effect of MC.^112^

There are many painful experiences encountered by the child before, during, and after birth.^113^ MC, *if performed without anesthetic*, is one of these. Cortisol levels, heart rate, and respiration have registered an increase during and shortly after infant MC.^114^^,^^115^ Adequate anesthesia is essential for pain management during MC at any age. Most MC procedures can be performed under local anesthesia. General anesthesia involves risks, is usually unnecessary, and is falling out of favor. The AAP^5^ and CDC^2^ recommend local anesthesia for infant MC.

Frisch and Earp take issue with a study the CDC cited related to the issue of pain associated with the MC procedure, arguing that the figures cited from the study were inaccurate. The study objectively scored pain experienced by newborns when undergoing MC and concluded that “painless circumcision [by Gomco clamp] is possible in almost all newborns if it is performed during the first week of life.”^116^ It is regrettable that there were indeed some errors in the figures reported in the study—in the abstract of the article, 6.5% of infants 1 week old or younger were reported to have pain scores of 2 or greater, whereas the source table in the main body of the article reports the figure of 6.7% and the raw data indicate the figure should actually be 7.1%. However, the error is trivial, resulting in a minor difference of up to 0.6 percentage points and thus does not negate the study's overall conclusion.

In addition, Frisch and Earp highlight that infants may also experience pain from administration of the anesthesia itself before the MC. Pain does occur during injection of local anesthetics, but it can be reduced by prior application of readily available topical anesthetic creams containing lidocaine and prilocaine (EMLA, or the more potent LMX4). In a clinical trial, application of EMLA cream 2 hours prior to Plastibell MC resulted in near absence of evidence of pain during and for 4 hours after infant MC, by which time nerves at the ablation site would have died, meaning a pain-free MC.^117^ Furthermore, we contend that any pain associated with injection of local anesthetic is no greater than pain incurred with injection of a vaccine.

While infants may experience pain from administration of a local anesthetic before circumcision, the pain can be reduced by prior application of topical anesthetic creams.

A small telephone survey, misconstrued by Frisch and Earp, actually found parents' (subjective) perception of level of discomfort among infants circumcised at 4–167 days of age (mean, 41.7 days) was mild in 84% of cases, moderate in 11%, and severe in only 5%.^118^ The average discomfort score for MC was less than for other simple ambulatory pediatric procedures evaluated in the study. Similarly, Frisch and Earp summarize results of another telephone survey^119^ by stating that “71% of parents reported varying degrees of circumcision-related pain in their infants … up to six weeks after surgery.” When analyzing the study's results in detail, however, one finds that only about 2% of parents whose sons were circumcised using a Gomco clamp reported “more than acceptable pain” (1.5%) or “much more pain” (0.9%). In comparison, 29% reported “no pain,” 15% reported “minimal pain,” and 53% reported “acceptable pain.” For Plastibell MC, these figures were 32% (no pain), 11% (minimal pain), 50% (acceptable pain), 3.2% (more than acceptable pain), and 3.8% (much more pain).^119^

Men circumcised as adults are well placed to communicate MC-related pain. In the 3 large RCTs of adult MC conducted in sub-Saharan Africa, only 0.8%,^120^ 0.3%,^121^ and 0.2%^122^ of men reported severe pain.

In the 3 large RCTs of adult male circumcision conducted in sub-Saharan Africa, less than 1% of men reported severe pain with the procedure.

## COMPLICATION RATES AFTER CIRCUMCISION

Frisch and Earp speculate about adverse MC-related events after discharge from hospital and over the long-term. They considered meatal stenosis to be “particularly worrying,” but misconstrue data on its prevalence, which, as noted earlier in this article, we argue will not affect “between 5% and 20% of boys undergoing non-therapeutic circumcision.” A recent study by Frisch and Simonsen found meatal stenosis incidence in Denmark to be very much lower than those figures and higher in uncircumcised than in circumcised elderly men,^123^ possibly contributed by lichen sclerosus. A critical evaluation of the literature suggests prevalence is in the order of 0.01%–1%, with a similarly low frequency among both circumcised and uncircumcised boys.^13^^,^^20^^,^^44^^,^^124^

Frisch and Earp also point to findings of retrospective case study that 4.7% of cases operated on in the pediatric surgery department of the MassGeneral Hospital for Children between 2003 and 2007 were for late complications related to newborn MC.^125^ Since that hospital serves the wider Boston area and receives cases following MC elsewhere, the sample is not representative. Besides vaccination, newborn MC is the most common pediatric procedure among males in the United States. Frisch and Earp concede that the “total number of circumcisions [that these figures relate to are] unknown.” In contrast, a recent study of 95,046 elective MCs from 2004 to 2013 in ambulatory surgery centers of 43 U.S. tertiary care pediatric hospitals found only 0.1% underwent a second ambulatory procedure within the first 7 days, being higher for older boys than for infants.^126^

## OTHER MC POLICY STATEMENTS

The draft CDC recommendations advocate informing parents of newborn boys and adolescent and adult men about the benefits and risks of MC,^3^ and the accompanying technical report^2^ refers to an analysis that finds the benefits of MC exceed the risks.^8^ The AAP policy also concluded that benefits of MC exceed the risks. Policy statements from Australian, British, and Dutch medical bodies, however, are more conservative or even negative about MC. Frisch and Earp point to these differences as a “lack of international agreement with the U.S. view,”^6^ but they fail to mention that none of these other bodies go to the level of claiming that MC detracts from sexual pleasure or function, oppose MC in high-HIV prevalence countries, or recommend that MC should be legislated against in their own countries. One negative policy, by the Royal Australasian College of Physicians, even maintains a relatively balanced view on MC, stating that^127^:

1. It is reasonable for parents to weigh the benefits and risks of circumcision.
2. To make the decision whether or not to circumcise their sons, the medical attendant is obliged to provide accurate, unbiased, and up-to-date information on the risks and benefits of the procedure.
3. Parental choice should be respected.
4. The operation should be undertaken in a safe, child-friendly environment by an appropriately trained competent practitioner, capable of dealing with the complications and using appropriate analgesia.

And while the CPS (Canada) newborn MC position statement^38^ does not recommend routine MC of every newborn male, it does acknowledge that “there may be a benefit for some boys in high-risk populations and circumstances where the procedure could be considered for disease reduction or treatment.”

The policy statements by the CDC and AAP have raised the bar. Policy statements on MC by medical bodies should follow their lead and rely on a thorough evaluation of the medical evidence to support their conclusions.

Policy statements on male circumcision by medical bodies should rely on medical evidence.

## CONCLUSION

We find major shortcomings in the criticisms by Frisch and Earp of the CDC's draft MC recommendations. In summary, the current scientific evidence shows that MC provides protection against numerous adverse medical conditions and infections, and the benefits of the procedure, including cost-savings over the long-term, greatly exceed risks, with benefits found in both poor and wealthy countries such as the United States. In addition, MC has no adverse effect on sexual function, sensitivity, or pleasure, nor is there reliable evidence for any long-term adverse psychological effect of MC. Furthermore, complication rates following the procedure are low, especially following early infant MC. Finally, pain that may be associated with the procedure during the first week of life can be negligible when local anesthesia is used.

Criticisms of the AAP and CDC policies by MC opponents have been consistently exposed as flawed (AAP policy^42^^,^^128^^–^^131^; CDC policy^132^^,^^133^). Convincing arguments have been made that it would be unethical to withhold information about the risks and benefits of MC from parents of boys.^130^^–^^132^^,^^134^^,^^135^ as recommended by the AAP and CDC. Curiously, those who condemn parent-approved infant MC are not as quick to condemn procedures that provide no medical benefit to children (e.g., cosmetic orthodontia, correction of harelip, surgery for tongue-tie, treatment of dwarfism by growth hormone injections, and surgery for removal of supernumerary digits).^135^ Why then do some regard MC as controversial?^135^ Article 24(1) of the United Nations Convention on the Rights of the Child states, “States Parties recognize the right of the child to the enjoyment of the highest attainable standard of health” and “shall strive to ensure that no child is deprived of his or her right of access to health care services.”^136^ Therefore, we assert that the CDC's draft MC recommendations do nothing more than advocate appropriately the right of male infants, children, adolescents, and adults to access health care services with medical benefits—that is, MC—and that adoption of the draft CDC recommendations into formal policy should improve public health in the United States.

Convincing arguments have been made that it would be unethical to withhold information about the risks and benefits of male circumcision from parents of boys.

Adoption of the draft CDC recommendations into formal policy should improve public health in the United States.

## Notes

**Peer Reviewed**

**Competing Interests:** Dr. Morris reports that he is a member of the Circumcision Academy of Australia, a government registered incorporated association whose Constitution states that it is “a non-profit organization” whose objectives are to “educate health professionals and the general public about male circumcision, including but not limited to the benefits, the risks and methods of male circumcision” and “to promote ease of access and affordability of male circumcision in Australia.” Dr. Krieger reports that he performs male circumcision in his clinical practice as a urologist, outside the submitted work. In addition, Dr. Krieger has a patent pending for a male circumcision device. Dr. Klausner has nothing to disclose.
